# Nucleocapsid Specific Diagnostics for the Detection of Divergent SARS-CoV-2 Variants

**DOI:** 10.3389/fimmu.2022.926262

**Published:** 2022-06-10

**Authors:** Ariel Isaacs, Alberto A. Amarilla, Julio Aguado, Naphak Modhiran, Eduardo A. Albornoz, Alireza A. Baradar, Christopher L. D. McMillan, Jovin J. Y. Choo, Adi Idris, Aroon Supramaniam, Nigel A. J. McMillan, David A. Muller, Paul R. Young, Trent M. Woodruff, Ernst J. Wolvetang, Keith J. Chappell, Daniel Watterson

**Affiliations:** ^1^School of Chemistry and Molecular Biosciences, University of Queensland, St. Lucia, QLD, Australia; ^2^Australian Institute for Biotechnology and Nanotechnology, University of Queensland, St. Lucia, QLD, Australia; ^3^School of Biomedical Sciences, Faculty of Medicine, University of Queensland, St. Lucia, QLD, Australia; ^4^Menzies Health Institute Queensland, School of Pharmacy and Medical Sciences, Griffith University, Gold Coast, QLD, Australia; ^5^Australian Infectious Disease Research Centre, University of Queensland, Saint Lucia, QLD, Australia

**Keywords:** SARS-CoV-2, nucleocapsid, nanobody, immunoassays, immunofluorescence, immunoplaque assay, diagnostics

## Abstract

Since the start of the COVID-19 pandemic, multiple waves of SARS-CoV-2 variants have emerged. Of particular concern is the omicron variant, which harbors 28 mutations in the spike glycoprotein receptor binding and N-terminal domains relative to the ancestral strain. The high mutability of SARS-CoV-2 therefore poses significant hurdles for development of universal assays that rely on spike-specific immune detection. To address this, more conserved viral antigens need to be targeted. In this work, we comprehensively demonstrate the use of nucleocapsid (N)-specific detection across several assays using previously described nanobodies C2 and E2. We show that these nanobodies are highly sensitive and can detect divergent SARS-CoV-2 ancestral, delta and omicron variants across several assays. By comparison, spike-specific antibodies S309 and CR3022 only disparately detect SARS-CoV-2 variant targets. As such, we conclude that N-specific detection could provide a standardized universal target for detection of current and emerging SARS-CoV-2 variants of concern.

## Introduction

The SARS-CoV-2 virus has caused a global pandemic of unprecedented magnitude, with over 450 million infections and 6 million deaths as of March 2022. To date, several vaccine technologies targeting the spike glycoprotein have been developed and have demonstrated high efficacies against severe illness and hospitalization (1–4). Despite this, due to the high mutability of RNA viruses, several variants have emerged that are capable of evading immune detection and binding by previously characterized antibodies (5–7). Not only does this have significant detrimental effects on vaccine efficacy, but it also poses a significant hurdle for diagnostics and assays that make use of such antibodies.

Most recently, the omicron SARS-CoV-2 strain has emerged and is currently being established as the dominant strain worldwide. This strain possesses many mutations throughout its genome, with many localized within the spike glycoprotein. In comparison to the ancestral strain, there are 15 amino acid substitutions present with the receptor binding domain (RBD) and 4 substitutions, 6 deletions and 3 insertions within the N-terminal domain (NTD) of spike (8). Collectively, these have resulted in the loss of binding of many antibodies used for immunoassays and diagnostics. Given this, alternative targets for *in vitro* assays must be established that are less amenable to virus mutation.

In addition to the spike glycoprotein, the SARS-CoV-2 virus possesses three other structural proteins including the membrane (M), envelope (E) and nucleocapsid (N). The nucleocapsid protein of SARS-CoV-2 is present in high quantities within virions and cells during infection and is critical for viral replication and protein packaging. During virus production, the N protein binds RNA molecules and forms RNA-protein complexes and through interaction with the M protein recruits the viral genome to newly-formed virions (9, 10). The N protein structure consists of a N-terminal domain (NTD) responsible for RNA binding and a C-terminal domain (CTD) involved in dimerization. Both of these domains are flanked by intrinsically disordered regions (IDRs) (9). Recent analyses have shown that mutations within SARS-CoV-2 N have predominately accumulated within the IDRs, likely due to the functional importance of the CTD and NTD (11). This is exemplified by the omicron SARS-CoV-2 strain, where 3 substitutions and 3 deletions were discovered all within the IDRs of N.

Given the conserved nature of the CTD and NTD and its high expression level during infection, N poses as an attractive target for detection in immunoassays and diagnostics. To this end, two N-specific nanobodies were recently isolated and structurally characterized that bind either the CTD or NTD (11–13). In this body of work, we demonstrate the use of these nanobodies in detecting SARS-CoV-2 variant infection *via* a comprehensive analysis of immunoassays including cell-based ELISAs, immunoplaque assays (IPA), immunofluorescence assays (IFA), western blot and immuno-detection of infected tissue.

## Materials and Methods

### Nanobody Design & Expression

Two N-specific nanobodies, C2 and E2, were previously described targeting the NTD and CTD of N, respectively (11–13). Nanobody sequences were acquired from protein data bank (PDB 7N0I and 7N0R) and ordered as synthetic gene blocks from Integrated DNA Technologies. Sequences were cloned into mammalian expression vectors containing a dimeric Fc tag *via* inFusion cloning (TakaraBio), as previously described (14). Plasmid DNA sequences encoding C2-Fc and E2-Fc were transfected and expressed in the ExpiCHO-S (ThermoFisher) expression system as per manufacturer’s guidelines. Briefly, ExpiCHO cells were seeded at a density of 1 ×10^6^ cells/mL and transfected with 1 μg plasmid DNA per 1 mL of cells. The following day, cultures were supplemented with ExpiCHO Feed and Enhancer as per manufacturer’s instructions. Seven days post-transfection, cell culture supernatant containing secreted nanobodies was harvested *via* centrifugation at 4800 ×g for 30 mins before filter sterilization (0.22 μm). Nanobody Fc constructs were purified by passing supernatant through a HiTrap Protein A HP (GE Healthcare) column followed by extensive washing with wash buffer (25 mM Tris, 25 mM NaCl, pH 7.4). Nanobodies were eluted using low pH elution buffer (100 mM sodium citrate, 150 mM NaCl, pH 3) and neutralized with an equal volume of 1.5 M Tris-HCl pH 8.8. Nanobodies were then concentrated and buffer exchanged to PBS using a 30 MWCO centrifugal concentrator (Merck Amicon).

### Viral Isolates

In this study, we made use of three low passages of SARS-CoV-2 viral isolates. An Ancestral strain: hCoV-19/Australia/QLD02/2020 (GISAID accession ID, EPI_ISL_407896), collected on 30^th^ of January 2020; Delta variant: hCoV-19/Australia/QLD1893C/2021 (GISAID accession ID EPI_ISL_2433928) collected on 5^th^ of April 2021; Omicron variant: hCov-19/Australia/NSW-RPAH-1933/2021 was isolated as previously described (15). All variants were propagated (passages 3) on VeroE6-TMPRSS-2.

### Cell-Based ELISA

Vero E6 cells were cultured and seeded at a density of 7 × 10^4^ cells per well of a 96 well plate in DMEM supplemented with 10% FCS. The following day, media was replaced to DMEM supplemented with 2% FCS and cells were infected with SARS-CoV-2 viral strains at a MOI of 0.1. Infected cells were incubated at 37°C with 5% CO_2_ for a further 36 hours before fixation with 80% acetone solution in PBS for 1 hour at -20°C. Fixed plates were allowed to dry and then blocked for 1 hour with 1X KPL solution (SeraCare) diluted in PBS with 0.1% Tween20 (PBST). Nanobodies or antibodies were serially titrated in block buffer and added onto fixed cells for 1 hr at 37°C. Plates were then washed three times in PBST before adding horse radish peroxidase (HRP)-conjugated goat anti-human secondary antibody (Life Technologies) diluted 1:2500 in block buffer. Plates were incubated for 1 hr at 37°C and washes were repeated. Plates were then revealed by adding TMB chromogen solution (Life Technologies) for 5 mins at room temperature. Reactions were stopped with 1 M H_2_SO_4_ and plate absorbances were read at 450 nm. Data was plotted with background binding of secondary only subtracted and a one-site specific model fitted on GraphPad Prism 9.

### Immunofluorescence Assay

Vero E6 cells were plated on glass coverslips at a density of 3 × 10^5^ cells/well. The following day, cells were infected with SARS-CoV-2 viral strains at an MOI of 0.1. Infected cells were incubated at 37°C with 5% CO_2_ for a further 36 hours before fixation with 4% paraformaldehyde for 1 hour. Fixed cells were washed with PBS and then blocked with PBS with 0.5% bovine serum albumin and 0.2% gelatin from cold water fish skin (Sigma Aldrich) (PBG) for 1 hour at room temperature. Concurrently, cells were permeabilized with 0.2% TritonX-100. Primary antibody or nanobody was diluted to 5 μg/mL in PBG and incubated on cells for 1 hr at room temperature. Cells were then washed three times in PBS before incubation with goat anti-human 647 secondary antibody diluted 1:1000 in PBG for 45 mins at room temperature in the dark. Coverslips were washed three times with PBS. Cell nuclei and cytoskeleton were stained with Hoescht stain (1 μg/mL) and phalloidin-fluorescein isothiocyanate (50 μg/mL, Sigma Aldrich), respectively, for 20 mins at room temperature. Cells were washed one final time before mounting. Immunofluorescence images were acquired using a Zeiss AxioScan Z1 Fluorescent Imager. The number of positive cells per region of interest for SARS-CoV-2 markers tested was analysed by the imaging software CellProfiler, using the same pipeline for each sample in the same experiment.

Infection of mouse brain tissue was conducted prior as previously described (16). Immunofluorescence staining protocols and imaging of OCT-embedded cryopreserved 4 μm-thick mouse brain sections were performed using identical protocols as for the 2D cell cultures.

### Immunoplaque Assay

Immunoplaque assays were conducted as previously described (17). Briefly, approximately 5×10^4^ cells per well of Vero E6 were seeded in 96 well plates and incubated overnight until they reached 100% confluency. Samples were serially diluted 10-fold in DMEM supplemented with 2% FCS and P/S, and the monolayer was incubated with 25 μL of the sample. After 30 min of incubation, 175 μL of overlay medium was added to each well. Then, 22 hrs after infection, the overlay was removed, and the monolayer was fixed with cold 80% acetone in PBS and kept for 30 min at -20°C. The plate was fully dried for 2 hrs and probed for viral proteins. Here, plates were first blocked for 60 min at 37°C with 100 μL blocking solution (KPL Milk Diluent/Blocking Solution Concentrate, Sera care, USA). Plates were then incubated with 50 μl/well of primary antibody at 1 μg/mL in blocking solution for 1 hr at 37°C, followed by five washes with PBS containing 0.05% Tween 20 (PBS-T). Plates were then incubated with 50 μl/well of a secondary fluorophore-conjugated antibody (IRDye, LI-COR, USA) at 1 μg/mL in blocking solution for 1 hr at 37°C, followed by five washes with PBST. Fully dried plates were scanned using the LI-COR Biosciences Odyssey Infrared Imaging System (Odyssey CLX, Li-COR, USA). The titer was expressed as focus forming units per millilitre, or FFU/mL.

### Western Blot

Vero E6 cells were plated at a density of 1.5×10**^6^** cells/well. The following day, cells were infected with SARS-CoV-2 viral strains at an MOI of 0.1. At 36 hours post infection, the monolayers were washed three time with cold PBS and then 300 μl/well of 2% SDS was added to each well and incubated for 5 mins. Cell lysates were sonicated and run on a Mini-Protean^®;^ precast gel (BioRad). SDS-PAGE gel was fixed in 20% ethanol for 5 mins and then transferred to nitrocellulose membrane using iBlot 2.0 (ThermoFisher). Membrane was blocked overnight at 4°C in 1X KPL (SeraCare) in PBST (block buffer). A mix of primary antibody at 2 μg/mL and anti-GADPH (Novus Biologicals) mouse antibody diluted 1:1000 in block buffer was added for 1 hr at room temperature. Blots were washed three times in PBST. A mix of anti-human IR800 or anti-mouse 680 (Millennium Sciences) secondaries diluted 1:5000 in block buffer was then added for 1 hr at room temperature in the dark. Blots were washed as before and then imaged on an Odyssey LICOR system.

## Results

**Figure 1:**
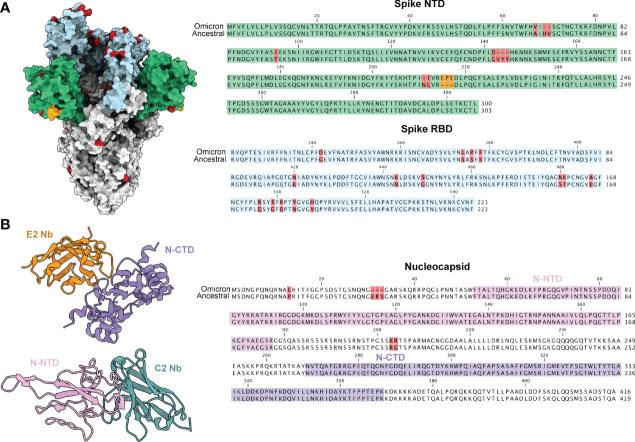
SARS-CoV-2 variant mutations are predominately localized to the spike glycoprotein. **(A)** A model of the ancestral SARS-CoV-2 spike glycoprotein (PDB 6VXX) with NTD coloured in green and RBD coloured in light blue. Mutations present in the omicron variant (7QO7) are highlighted in red and insertion in orange. Sequence alignments of NTD and RBD are shown to the right with the same coloring scheme. **(B)** Structures of E2 (orange, PDB 7N0I) and C2 (turquoise, PDB 7N0R) nanobodies complexed with either the CTD (purple) and NTD (pink) of the nucleocapsid protein. The NTD and CTD of N are coloured in a sequence alignment to the right with mutations present in omicron coloured in red.

**Figure 2:**
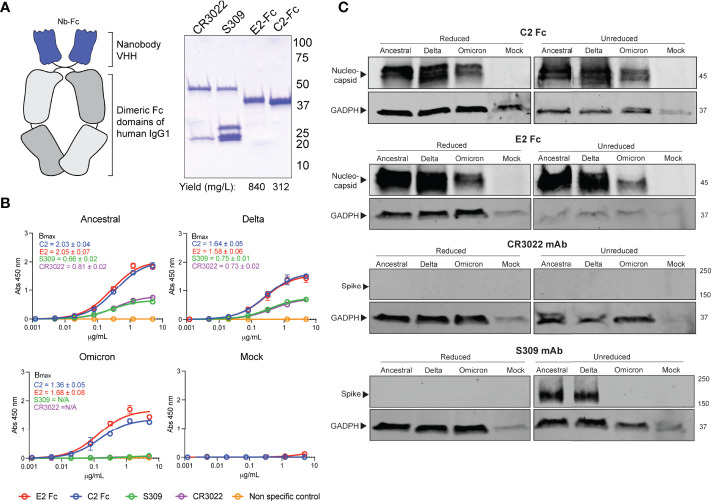
Detection of SARS-CoV-2 variants by N-specific nanobodies. **(A)** C2 and E2 nanobodies were formatted as dimeric Fc fusion proteins for ease of purification and detection. Coomassie stained SDS-PAGE shows highly pure products. **(B)** Differential detection of viral antigens in fixed cell-based ELISA against infected Vero E6 cells. Assays were conducted twice with n = 2. Bmax values with standard error are shown for each variant as calculated by Prism Graphpad 9 software. **(C)** Detection of viral antigens in infected cell lysates in western blots ran under reducing (+DTT) or non-reducing conditions. Housekeeping protein GADPH was used as a positive control. Molecular weights are shown to the right in kDa.

Current immunoassays predominately make use of spike specific antibodies for virus detection. The omicron strain of SARS-CoV-2 has acquired a number of mutations, with the majority localized within the RBD and NTD of spike (**Figure 1**). This has resulted in ablation of majority of known antibody epitopes and therefore poses a hurdle for spike-specific diagnostic assays (5–7). The N protein of SARS-CoV-2 poses as an alternative target for immunoassays, made possible through targeting the conserved NTD and CTD. Here, we outline the use of two previously characterized nanobodies, C2 and E2, that target the NTD and CTD of N, respectively. For ease of detection & purification, we expressed C2 and E2 with a dimeric human Fc tag, allowing purification *via* standard protein A affinity as well as detection using conventional anti-human secondary antibodies (**Figure 2A**). We observed highly pure C2 and E2 preparations with high yields of 312 and 840 mg/L of ExpiCHO supernatant, respectively.

We first sought to confirm the binding of the nanobody constructs *via* an infected cell-based ELISA (**Figure 2B**). Here, Vero E6 cells were infected with either an ancestral SARS-CoV-2 isolate, the delta strain or the omicron variant. We observed that both C2 and E2 nanobodies were able to detect infected cells in a dose-dependent manner for all strains. Interestingly, the ELISA B_max_ values for N-specific nanobodies was substantially higher than spike-specific mAbs for all strains tested (**Figure 2B**). Given that detection limits are dependent on affinity and epitope availability, it is likely that C2 and E2 are more sensitive than S-specific mAbs due to the high abundance of N during infection. In contrast to C2 and E2, spike-specific mAbs CR3022 and S309 were not able to detect omicron spike proteins in the cell-based ELISA. This may be in part due to the reduced affinities of these mAbs against omicron spike, which may further exacerbate detection and assay sensitivity. Similar results were also observed in western blots (**Figure 2C**). Here, C2 and E2 were able to detect N from all SARS-CoV-2 strains in infected cell lysates under both reducing and non-reducing conditions. In contrast, the CR3022 mAb could not detect spike and S309 mAb could only detect the ancestral and delta strain spike proteins under non-reducing conditions.

**Figure 3:**
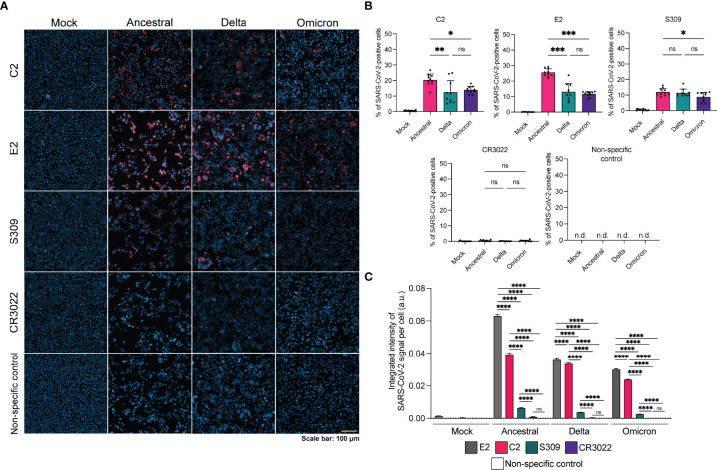
Detection of SARS-CoV-2 variants by N-specific nanobodies *via* IFA. **(A)** Representative IFA images of infected Vero E6 cells stained with antibody or nanobody (red) and Hoescht stain (blue). Non-specific control is a Nipah F specific mAb 5B3. **(B)** Quantification of percentage of SARS-CoV-2 positive cells per region of interest (n=10) for each staining method. **(C)** Integrated intensity of SARS-CoV-2 signal per cell per strain for each staining method. Number of cells analyzed per condition is as follows: mock infected, E2 n=36,393; mock infected, C2 n=34,712; mock infected, S309 n=36,360; mock infected, CR3022 n=35,700; mock infected, non-specific n=37,166; ancestral, E2 n=9621; ancestral, C2 n=9699; ancestral, S309 n=18,088; ancestral, CR3022 n=7650; ancestral, non-specific n=24,307; delta, E2 n=19,517; delta, C2 n=18,359; delta, S309 n=23,476; delta, CR3022 n=19,104; delta, non-specific n=27,871; omicron, E2 n=19,359; omicron, C2 n=29,133; omicron, S309 n=11,565; omicron, CR3022 n=26,210; omicron, non-specific n=22,892. Statistics were performed using a one-way ANOVA with Tukey’s multiple comparisons test where *, p < 0.05, **, p < 0.005, ***, p < 0.001, ****, p < 0.0001, ns, non-significant and n.d., not detected.

Next, we sought to evaluate the use of C2 and E2 in IFA (**Figure 3**). Vero E6 cells were infected with SARS-CoV-2 variants, fixed and permeabilized prior to staining. We observed that C2 and E2 were able to detect infection for all variants, with higher levels of positive cells per region of interest for the ancestral strain in comparison to delta or omicron (**Figure 3B**). Spike-specific mAb S309 could also detect all three variants, albeit with reduced signal relative to C2 and E2. Interestingly, CR3022 did not detect any of the variants in the IFA despite its ability to bind the ancestral and delta strains in a fixed cell ELISA (**Figure 2B**). The integrated signal intensity per cell for each staining method was also examined. For all variants, the signal intensity per cell was highest for E2 followed by C2 (**Figure 3C**). Spike-specific signal intensity per cell as measured by S309 was significantly lower than N-specific and CR3022 signal was non-significant compared to the non-specific control.

**Figure 4:**
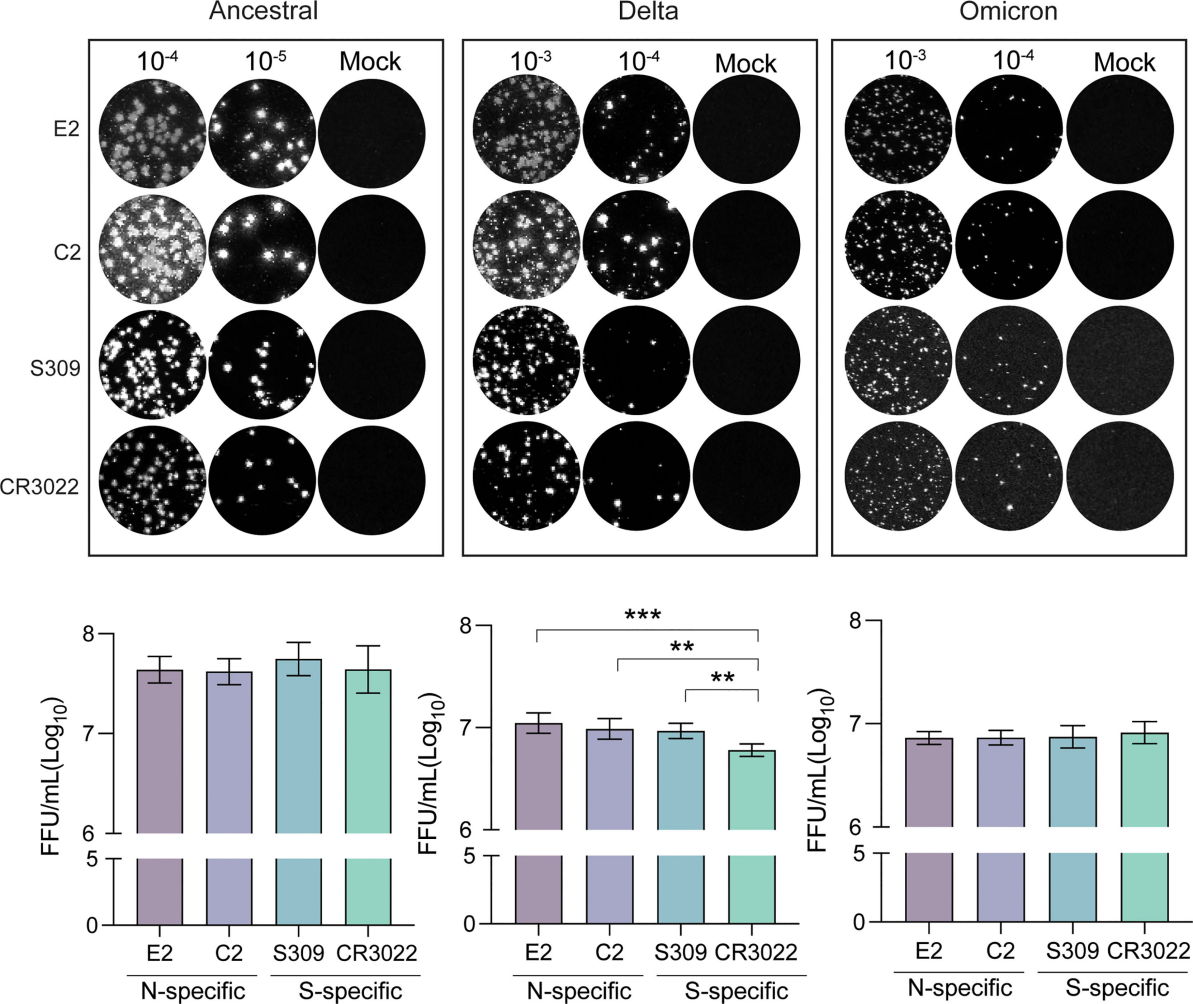
Immunoplaque assays using N-specific immunostaining. Representative dilutions for each variant and staining method are shown. Titers for each variant are shown below calculated from analyses in triplicate. Statistical analysis was conducted using an ordinary one-way ANOVA with Tukey’s test where ***, p < 0.0005 & **, p < 0.005.

As new SARS-CoV-2 variants emerge, it is important to have a standardized method for quantification of viral titer and testing of virus neutralization. We have previously outlined an immunoplaque assay (IPA) method that made use of spike-specific antibodies for staining of viral plaques (17). In light of the emergence of the omicron strain, immunostaining techniques that target conserved viral antigens would allow for a more universal application of this method. To this end, we tested C2 and E2 nanobodies as reagents for identifying viral plaques *via* IPA (**Figure 4**). Interestingly, we found that all antibodies used for immunostaining were able to detect plaques, despite differential detection capacities in previous assays. Of note, omicron plaque sizes were considerably smaller in comparison to delta and ancestral strains. As such, brightness levels had to be substantially increased for spike specific detection by S309 and CR3022, but not for C2 and E2. We estimated viral titers of different strains using each antibody and found that similar titers for C2, E2 and S309, however significantly lower titers were detected using CR3022 for the delta variant.

**Figure 5:**
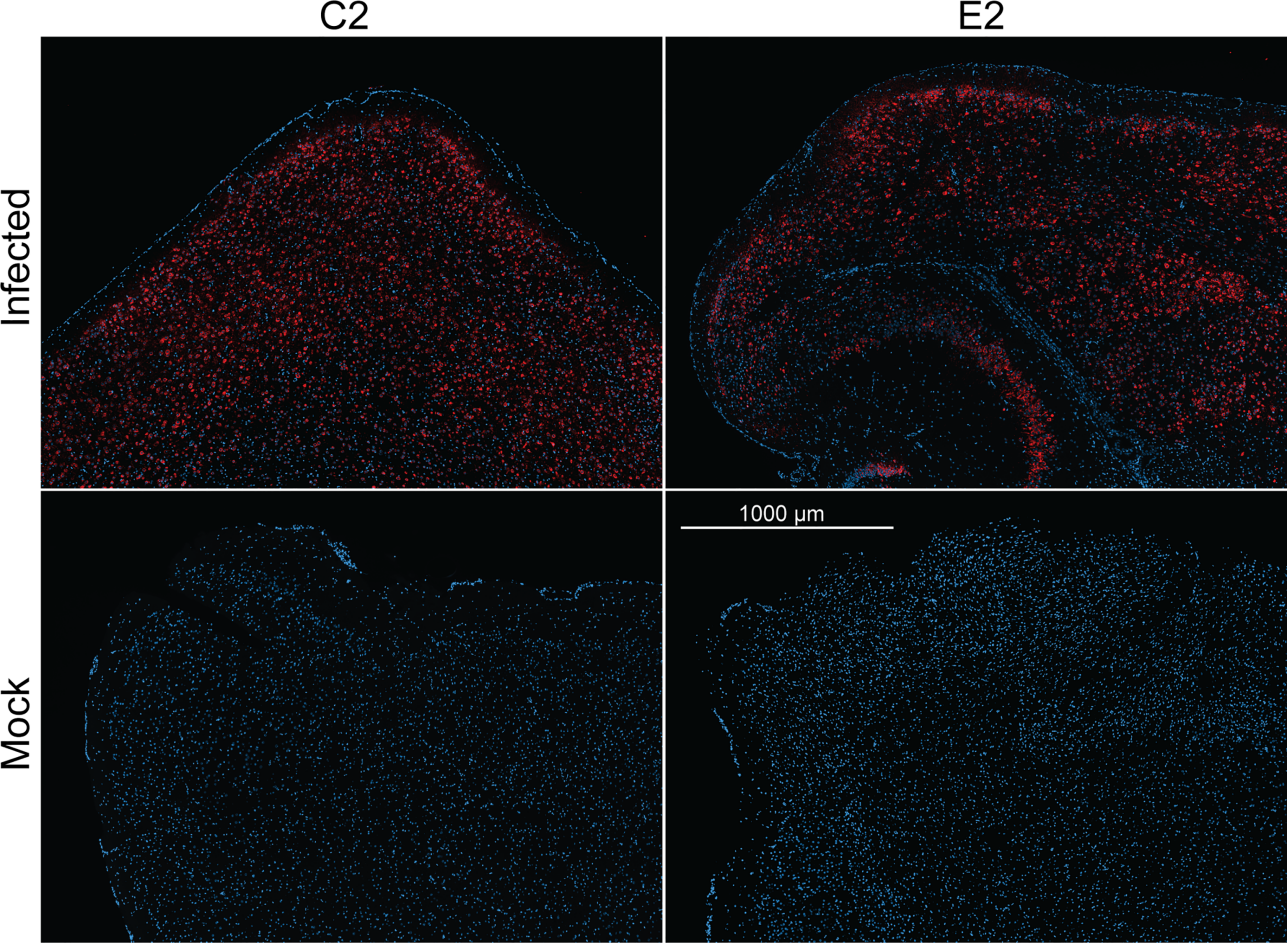
Immunofluorescence staining of naïve or infected brain tissue. K18-hACE2 mice were previously challenged with an ancestral strain of SARS-CoV-2 (hCoV-19/Australia/VIC01/2020). Paraffin embedded sectioned brain tissue was then stained with either C2 Fc or E2 Fc nanobodies (red) & DAPI (blue) to detect N antigen and nucleus, respectively.

Finally, we evaluated the use E2 and C2 nanobodies in detecting SARS-CoV-2 in infected brain tissue (**Figure 5**). Previously, K18-hACE2 mice were challenged with an ancestral strain of SARS-CoV-2 (hCoV-19/Australia/VIC01/2020). Brain tissue samples were acquired and embedded in paraffin prior to immunofluorescence staining using C2 and E2. Here, we found that infected cells could be detected to a high degree with both C2 and E2, demonstrating their use in staining of infected tissue samples.

## Discussion

Throughout the COVID-19 pandemic, several SARS-CoV-2 variants have emerged. The current dominant strain is the omicron variant, which harbors several immune-evading mutations within the spike glycoprotein (5–7). Several studies have demonstrated the detrimental effects of these mutations on vaccine immunity where spike is the dominant target (18–20). By extension, the emergence of new variants such as delta and omicron also provides a hurdle for immune assays that make use of spike-targeting monoclonal antibodies. Targeting a more conserved protein, such as the nucleocapsid, would allow for more universal applications of assays as new variants emerge.

This body of work highlights the use of N-specific nanobodies for detection of SARS-CoV-2 virus in various assays. Between omicron and the ancestral SARS-CoV-2 strains, there are 2 substitution mutations and three amino acid deletions within N, however these are located within the IDRs. Previous research has demonstrated that these regions are 20-fold more likely to acquire mutations across SARS-CoV-2 variants in comparison to the NTD and CTD, where the epitopes for C2 and E2 reside (11). Given this, E2 and C2 present as useful tools for universal SARS-CoV-2 detection. To this end, we aimed to quantitatively and qualitatively demonstrate the use of these reagents in infected cell-based ELISAs, western blot, immunofluorescence assays and immunoplaque assays.

We found that N-specific detection by C2 and E2 was sensitive and consistent across several assays. For spike-specific detection, we obtained some differential results between assays. Firstly, despite its ability to bind the omicron variant spike protein (21), we found that S309 mAb failed to bind in a fixed cell-based ELISA and western blot. This may be due to the reduced affinity of S309 to omicron (22). Alternatively, the S309 epitope in omicron spike may be sensitive to acetone fixation and SDS detergent. Similar results were obtained for a second spike-specific mAb CR3022, which failed to detect the omicron variant in cell-based ELISAs and did not detect any variant spike proteins in a western blot. In contrast, C2 and E2 nanobodies consistently detected all variants N proteins in both cell-based ELISAs and western blots of infected cell lysates. Similar results were reiterated in the immunofluorescence assay. Here, we qualitatively and quantitively demonstrated the ability of N-specific nanobodies to detect SARS-CoV-2 variants over spike-specific mAbs. In particular, a higher amount of positive cells as well as higher signal intensities per cell were observed for N-specific nanobodies in comparison to S309. This is explained by the high prevalence of N during infection (23–26), as well as the high affinity of the nanobodies to their respective epitopes. We were also able to demonstrate the utility of C2 and E2 in SARS-CoV-2 virus detection in mouse brain tissue. While not directly tested, C2 and E2 will likely also be able to detect omicron and delta strains in mouse tissue given their cross reactivity in all other assays.

Previously, we have developed an optimized IPA to quantify SARS-CoV-2 viral titers for use in neutralization assays (17). Here we develop on this work and demonstrate that N-specific nanobodies are able to detect SARS-CoV-2 ancestral, delta and omicron plaques with similar titers to that of S-specific mAbs. We observed that the omicron strain yielded lower viral titers in comparison to delta or the ancestral strains, which is consistent with previous reports of slower replication of omicron in Vero cell lines (27). Of note, CR3022 mAb was able to detect all three variants in the IPA, however it was less sensitive in omicron detection, leading to higher background levels. As such, we recommend the use of N-specific nanobodies for universal detection of plaques formed by SARS-CoV-2 variants.

There are several limitations to this study. Firstly, only variants of SARS-CoV-2 were investigated for binding and detection by C2 and E2 nanobodies in immunoassays. Previously, C2 and E2 were shown to cross-react to SARS-CoV N given the high sequence homology to SARS-CoV-2 N (~90%), yet this did not extend to other coronaviruses (12). Therefore, it is likely that these reagents may also be useful in SARS-CoV based immunoassays, yet this remains to be experimentally validated. Secondly, we did not conduct conventional ELISAs against recombinant N protein. However, the versatility of C2 and E2 across the several immunoassays described in this work, in addition to their previous use in surface plasmon resonance & MagPlex viral antigen assays by others (12), suggests that a conventional ELISA would be successful and of use in SARS-CoV-2 based diagnostics.

## Data Availability Statement

The original contributions presented in the study are included in the article/supplementary material. Further inquiries can be directed to the corresponding author.

## Ethics Statement

The animal study was reviewed and approved by MHIQ/12/20/AEC.

## Author Contributions

AIs, AA and DW conceptualized the project. AIs, AA, DW, NM, and JA developed methodology. AIs, AA, and JA verified replication and reproducibility. AIs, AA, and JA applied formal techniques to analyze datasets. AIs, AA, JA, NM, EA, and AB conducted the research investigation process. CM, JC, AId, AS, NM, and DM provided resources and study materials. AIs wrote the original draft. All authors contributed to review and editing. DW, KC, TW, and EW provided supervision and oversight. DW, KC, PY, and EW acquired financial support.

## Funding

This work was funded by the Medical Research Future Fund (APP1202445-2020 MRFF Novel Coronavirus Vaccine Development Grant). JA was funded by a University of Queensland Early Career Researcher Grant (application UQECR2058457), a NHMRC Ideas Grant (APP2001408), and a Jérôme Lejeune Postdoctoral Fellowship. National Health and Medical Research Council (NHMRC) fellowship (2009957) to TMW. CSL Centenary Fellowship to DW.

## Conflict of Interest

The authors declare that the research was conducted in the absence of any commercial or financial relationships that could be construed as a potential conflict of interest.

## Publisher’s Note

All claims expressed in this article are solely those of the authors and do not necessarily represent those of their affiliated organizations, or those of the publisher, the editors and the reviewers. Any product that may be evaluated in this article, or claim that may be made by its manufacturer, is not guaranteed or endorsed by the publisher.
